# Role for A-Type Lamins in Herpesviral DNA Targeting and Heterochromatin Modulation

**DOI:** 10.1371/journal.ppat.1000071

**Published:** 2008-05-23

**Authors:** Lindsey Silva, Anna Cliffe, Lynne Chang, David M. Knipe

**Affiliations:** Department of Microbiology and Molecular Genetics, Harvard Medical School, Boston, Massachusetts, United States of America; University of California San Francisco, United States of America

## Abstract

Posttranslational modification of histones is known to regulate chromatin structure and transcriptional activity, and the nuclear lamina is thought to serve as a site for heterochromatin maintenance and transcriptional silencing. In this report, we show that the nuclear lamina can also play a role in the downregulation of heterochromatin and in gene activation. Herpes simplex virus DNA initiates replication in replication compartments near the inner edge of the nucleus, and histones are excluded from these structures. To define the role of nuclear lamins in HSV replication, we examined HSV infection in wild-type and A-type lamin–deficient (*Lmna* ^−/−^) murine embryonic fibroblasts (MEFs). In *Lmna* ^−/−^ cells, viral replication compartments are reduced in size and fail to target to the nuclear periphery, as observed in WT cells. Chromatin immunoprecipitation and immunofluorescence studies demonstrate that HSV DNA is associated with increased heterochromatin in *Lmna* ^−/−^ MEFs. These results argue for a functional role for A-type lamins as viral gene expression, DNA replication, and growth are reduced in *Lmna* ^−/−^ MEFs, with the greatest effect on viral replication at low multiplicity of infection. Thus, lamin A/C is required for targeting of the viral genome and the reduction of heterochromatin on viral promoters during lytic infection. The nuclear lamina can serve as a molecular scaffold for DNA genomes and the protein complexes that regulate both euchromatin and heterochromatin histone modifications.

## Author Summary

Transcription of eukaryotic genes is regulated by sequence-specific DNA-binding proteins that bind to the DNA as well as the structure of the chromatin associated with the specific gene. Posttranslational modification of histones plays a major role in the higher order structure of the chromatin and whether it serves as heterochromatin or inactive chromatin versus euchromatin or active chromatin. The nuclear lamina has been shown to promote the maintenance of heterochromatin in mammalian cells, but little is known about where heterochromatin is modulated. In this study, we find that the A-type lamins are required for the targeting of herpes simplex virus genomic DNA to the periphery of the infected cell nucleus and for the prevention or reduction of heterochromatin on the viral genome and transcriptional silencing of the viral genome. This raises the broader function of the nuclear lamina in the regulation of both euchromatin and heterochromatin. We propose that the nuclear lamina is a platform for the organization of chromatin remodeling and histone modification enzymes that regulate both euchromatin and heterochromatin.

## Introduction

Herpes simplex virus (HSV) undergoes productive infection through transcription and replication of its viral genome within the nucleus [1]. HSV gene expression involves the temporal expression of immediate-early (IE), early (E), and (L) genes [2] and the sequential remodeling of the infected cell nucleus by viral proteins [3]. One of the earliest demonstrations of the compartmentalization of nuclear processes, such as DNA replication, was the observation of replication compartment formation in the nuclei of HSV-infected cells. HSV replication compartments are the site of viral DNA replication, late gene transcription, and viral DNA encapsidation [4]. Replication compartments and parental genome complexes form at the nuclear periphery during early times of infection [4]–[6]. Furthermore, lamin A/C and the nuclear envelope emerin protein co-precipitated with the HSV DNA replication protein ICP8 in a proteomics study [7], suggesting an association of the replication compartment with the nuclear lamina and/or nuclear envelope. The nuclear lamina is disrupted at late times postinfection [8],[9], at least in part to allow access of the nucleocapsids to the inner nuclear envelope for budding and primary envelopment. However, nothing is known about the role of the nuclear lamina at early times postinfection.

A-type and B-type lamins are major components of the nuclear lamina that underlies the inner nuclear membrane and provides structural integrity to the nucleus [10]. The A-type lamins (lamins A, C, AD10, and C2) are expressed in differentiated cells and are encoded by the *LMNA* gene, whose products are encoded by transcripts generated by alternative splicing [11]. A-type lamins are found along the inner side of the nuclear envelope and within the nucleoplasm where they form a veil-like structure [12],[13]. Lamins are believed to function in higher order chromatin organization by acting as part of a molecular scaffold with integral membrane proteins to tether peripheral heterochromatin and chromatin remodeling complexes to the nuclear envelope [14],[15]. Evidence of lamin A/C function in chromatin organization has been provided by studies showing that mutations in the human *LMNA* gene lead to premature aging and progressive loss of heterochromatin [16],[17], indicating a role for the nuclear lamina in heterochromatin maintenance. Furthermore, immortalized mouse embryonic fibroblasts from *Lmna* ^−/−^ knockout mice exhibit alterations in nuclear envelope integrity, mislocalization of lamin binding proteins, and reduced peripheral heterochromatin [18],[19]. Targeting of genes to the nuclear periphery has been associated with gene silencing in several cases [20],[21]; however, in other cases the movement of active genes to the periphery is believed to be due to association of actively transcribing genes with nuclear pores [22],[23] and not the nuclear lamina. Hence, the role of nuclear targeting in regulation of gene expression remains to be fully defined. The available data suggest a role for the nuclear lamina in maintenance of heterochromatin and gene silencing.

Viruses have served as sensitive probes for the study of mechanisms of cellular processes, and chromatin plays an important role in regulation of HSV gene expression [24] . HSV DNA in the virion is not associated with histones [25]. As DNA enters the nucleus, cellular mechanisms attempt to silence the incoming genome through assembly of heterochromatin onto the DNA molecules, as first observed in transfected cells [26]. The HSV VP16 tegument protein plays a role in reducing histone association with viral DNA and in increasing the euchromatin modifications on the histones associated with viral genes [27],[28]. The HSV immediate-early (IE) ICP0 protein acts as an inhibitor of histone deacetylases [28]–[30]. In terms of the cellular mechanisms regulating chromatin structure, little is known about the nuclear location where chromatin modification takes place or is regulated beyond the role of the nuclear lamina in heterochromatin maintenance. This study provides evidence that lamin-dependent targeting of the HSV genome to the nuclear periphery is associated with a reduction of heterochromatin on viral lytic genes.

## Results

### Formation of viral replication compartments in *Lmna^−/−^* cells

**Figure 1:**
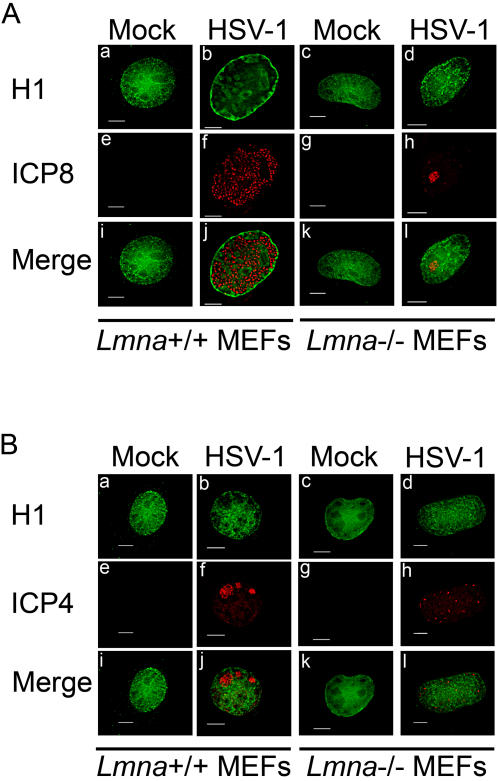
Replication compartments are reduced in size and histone H1 is not excluded from replication compartments in *Lmna^−/−^* MEFs. *Lmna^+/+^* (panels a, b, e, f, I, and j) and *Lmna* ^−/−^ (panels c, d, g, h, k and l) MEFs were either mock-infected or infected with HSV and fixed at 8 hpi (panel A) or 4 hpi (panel B). Fixed cells were processed for indirect immunofluorescence with antibodies specific for histone H1 (green) and either HSV-1 ICP8 (panel A; red) or ICP4 (panel B; red). Scale bars equal 5 µm.

Based on the localization of early viral replication compartments at the nuclear periphery and the co-precipitation of lamin A with the HSV ICP8 DNA replication protein, we hypothesized that the nuclear lamina plays a role in HSV transcription and DNA replication through recruitment of viral DNA and assembly of replication compartments at the inner nuclear membrane at early times postinfection. To define the role of lamin A/C in the formation of replication compartments in the nuclei of HSV-infected cells, we examined HSV infection in WT (*Lmna^+/+^*) and lamin A/C knockout (*Lmna* ^−/−^) immortalized mouse embryonic fibroblasts (MEFs) [18]. We first used immunofluorescence to define the role of lamin A/C in the assembly of viral replication compartments. *Lmna^+/+^* and *Lmna^−/−^* MEFs were either mock-infected or infected with HSV at a multiplicity of infection (MOI) of 10 PFU/cell, fixed at 8 hours post-infection (hpi), and stained with antibodies specific for the HSV ICP8 DNA replication protein and for histone H1. Mock-infected MEFs showed diffuse intranuclear histone H1 staining in both *Lmna^+/+^* and *Lmna^−/−^* cells, but the *Lmna^−/−^* cells showed reduced H1 staining near the nuclear envelope, consistent with reduced chromatin attachment to the nuclear envelope (Figure 1A, panels a and c). HSV-infected *Lmna^+/+^* MEFs contained intranuclear replication compartments, as evidenced by ICP8 staining at 8 hpi, which filled much of the interior of the nucleus and excluded histone H1 to the periphery and certain internal regions of the nucleus (Figure 1A, panels b, f and j). Surprisingly, *Lmna^−/−^* MEFs infected with the same amount of virus showed fewer cells containing replication compartments as detected by immunofluorescence, and the compartments observed were much smaller (Figure 1A, panels d, h and l). Second, the punctate ICP8 foci were more densely packed in the *Lmna* ^−/−^ cells than in the *Lmna* ^+/+^ cells (Figure 1A). Third, histone H1 was not segregated from the small replication compartments (same panels). Finally, the nuclei did not enlarge in *Lmna^−/−^* infected MEFs, in contrast to what was observed previously in primate cells [31] and in *Lmna^+/+^* MEFs (Figure 1A). Similar experiments looking at the IE ICP4 transactivator protein at 4 hpi also showed smaller replication compartments and a diffuse distribution of histone H1 in *Lmna^−/−^* MEFs as compared with *Lmna^+/+^* MEFs (Figure 1B). The smaller replication compartments observed in *Lmna^−/−^* cells were also observed at later times postinfection, e.g., 12 hpi (results not shown).

**Figure 2:**
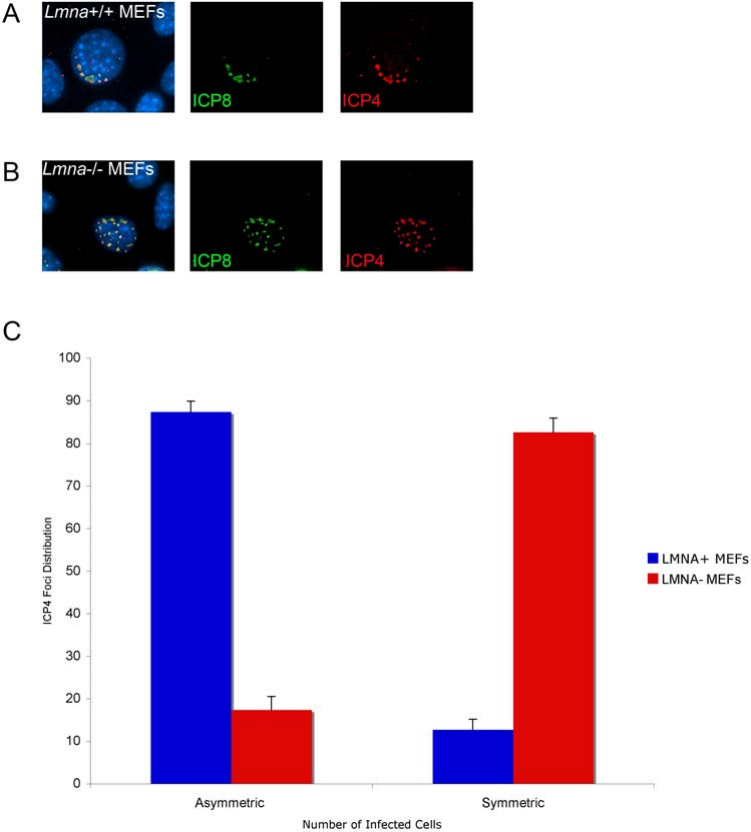
ICP4/8 foci fail to develop asymmetrically along the interior of the nuclear envelope in *Lmna^−/−^* cells. *Lmna^+/+^* and *Lmna^−/−^* MEFs were infected at 0.005 PFU/cell with HSV and fixed at 36 hpi following the onset of plaque formation. Cells were co-stained with ICP4 antibody (red), ICP8 antibody (green), and DAPI (blue). A) Example of *Lmna* ^+/+^ MEF showing asymmetric distribution of ICP4/ICP8 foci. B) Example of *Lmna* ^−/−^ MEF showing symmetric distribution of ICP4/ICP8 foci. C) Quantification of ICP4/ICP8 distribution patterns in *Lmna* ^+/+^ and *Lmna* ^−/−^ cells around plaques (n = 100 cells; 3 experiments).

To examine the role of lamin A/C in the intranuclear location of replication compartments, we infected *Lmna^+/+^* and *Lmna^−/−^* MEFs with HSV at a low MOI for 36 hours to allow for the development of discrete plaques. Plaques were smaller on the *Lmna^−/−^* cells and formed at an 8-fold lower efficiency on *Lmna^−/−^* cells as compared with *Lmna^+/+^* cells (L. Silva and D. Knipe, unpublished results). Previous studies had shown that in cells at the periphery of a developing plaque, replication compartments and genome complexes form along the inner nuclear envelope nearest the plaque [5],[6]. This was likely due to tethering of the viral genome and/or replication compartments at the nuclear periphery near the nuclear pore where the viral genome enters the nucleus. Immunofluorescence detection of the HSV immediate-early ICP4 protein was used to define early complexes as ICP4 is reported to associate with the parental viral genome [6], and detection of ICP8 was used to define early replication compartments [3]. In *Lmna^+/+^* MEFs at the edge of a plaque, developing replication compartments, as detected by ICP4 and ICP8 immunofluorescence, were assembled within the nucleus in an asymmetric distribution along one edge of the nucleus nearest the plaque (Figure 2A). However, this asymmetric ICP4 and ICP8 distribution was lost in the absence of lamin A/C (Figure 2B). To quantify this difference, we scored *Lmna^+/+^* and *Lmna^−/−^* MEFs according to the distribution of ICP4 foci. *Lmna^−/−^* MEFs displayed a 5-fold decrease in asymmetric ICP4 foci distribution as compared with *Lmna^+/+^* MEFs (Figure 2C). These results argued that a loss of lamin A/C may lead to an inability of the viral genomes to target to the nuclear periphery due to the absence of lamins or lamin-associated proteins that are required for recruitment of the incoming parental genomes, which ultimately develop into replication compartments.

### Viral gene expression is reduced in *Lmna^−/−^* cells

**Figure 3:**
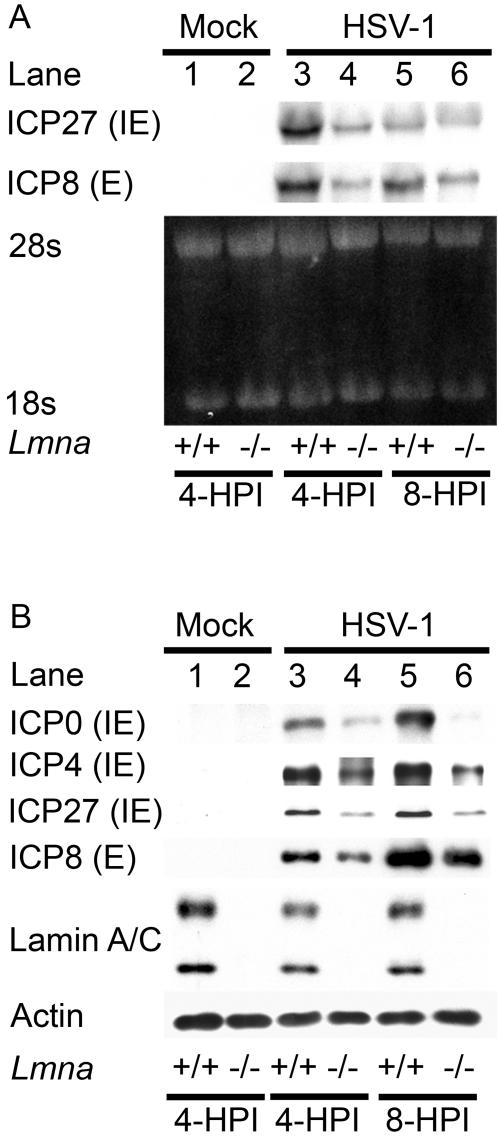
HSV immediate-early (IE) and early (E) gene expression is decreased in the absence of lamin A/C. *Lmna^+/+^* and *Lmna^−/−^* MEFs were mock-infected (lanes 1 and 2) or infected with HSV-1 at an MOI of 20 for 4 (lanes 3 and 4) or 8 hpi (lanes 5 and 6). At the times indicated, the cells were harvested RNA and protein analysis. A) Northern blot hybridization. Equivalent amounts of total RNA were resolved in a denaturing formaldehyde agarose gel stained with ethidium bromide. The mRNAs encoding ICP27 or ICP8 were detected by Northern blot hybridization using ^32^P-labeled DNA. B) Western blot analysis was performed with antibodies specific for the IE proteins ICP0, ICP4, and ICP27 and the E protein ICP8. rRNAs and actin were used as loading controls for the Northern and western blots, respectively.

The reduced levels of ICP8 immunofluorescence in HSV-infected *Lmna^−/−^* MEFs suggested that viral early gene expression was reduced. We therefore measured viral RNA and protein levels in *Lmna^+/+^* and *Lmna^−/−^* MEFs by northern and western blotting, respectively. Viral ICP27 (IE) and ICP8 (E) mRNA levels were reduced in *Lmna^−/−^* MEFs at 4 hpi (Figure 3A, lane 4). In addition, we observed that levels of ICP8 were reduced in *Lmna^−/−^* MEFs as early as 4 hpi and showed at least a 3-fold reduction at 8 hpi, as compared to the *Lmna^+/+^* MEFs (Figure 3B, lanes 4 and 6). These results argued that lamin A/C was required for early viral gene expression. Similar reductions in the immediate early proteins ICP0, ICP4, and ICP27 were observed in infected *Lmna^−/−^* MEFs as compared with *Lmna^+/+^* MEFs (Figure 3B, lanes 4 and 6). Thus, the earliest defect in viral gene expression in the *Lmna^−/−^* MEFs was reduced expression of IE genes.

### Increased Heterochromatin association with viral IE genes in *Lmna^−/−^* cells

**Figure 4:**
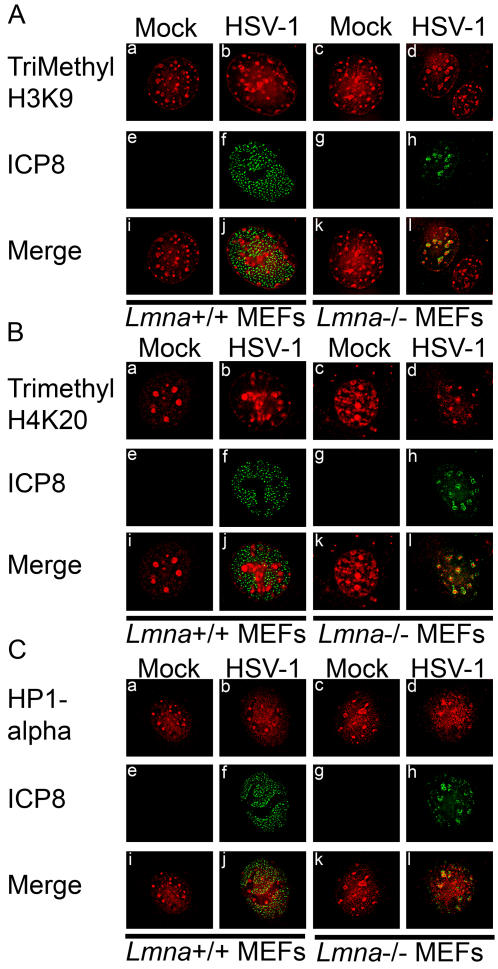
Heterochromatin exclusion from replication compartments during HSV-1 infection requires lamin A/C. *Lmna^+/+^* and *Lmna^−/−^* MEFs were either mock-infected or infected with HSV-1 at an MO of 20, fixed at 8 hpi, and stained with antibodies against ICP8 (green) and either trimethyl H3K9 (Panel A; red), trimethyl H4K20 (panel B; red), or heterochromatin protein 1α (panel C; red).

The increased levels of histone H1 co-localizing with replication compartments suggested that the reduced level of viral gene expression might be due to repressive effects of chromatin on viral genes. During productive infection with wild type virus, limited amounts of nucleosomes are associated with the viral genome [32],[33]. Histones that are associated with HSV DNA during productive infection have modifications that allow for active transcription [27]. Mature replication compartments exclude histone H1 [31] and cause the marginalization of the host chromatin [8]. To determine if heterochromatin was associated with viral replication compartments, we first examined mock- or HSV-infected cells using immunofluorescence to detect the trimethylated form of histone H3 lysine 9 (H3K9Me3) and histone H4 lysine 20 (H4K20Me3), both markers of heterochromatin. In *Lmna^+/+^* infected cells, heterochromatin was excluded from replication compartments (Figure 4A and 4B, panels b, f and j). In contrast, heterochromatin appeared coincident with the small replication compartments observed in HSV-infected *Lmna^−/−^* MEFs (Figure 4A and 4B, panels d, h and l), suggesting that heterochromatin was associated with replication compartments in these cells. Similar results were seen in immunofluorescence experiments detecting heterochromatin protein 1α (HP-1α), which recognizes and binds to trimethyl H3K9 (Figure 4C).

**Figure 5:**
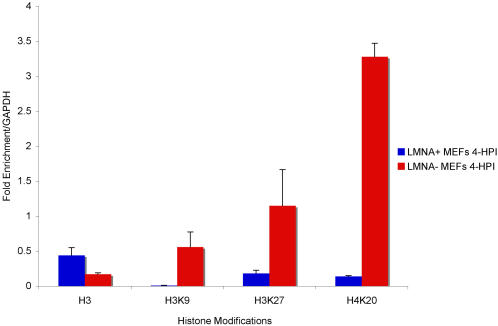
Heterochromatin on HSV-1 lytic promoter in the absence of lamin A/C. ChIP assays were performed using antibodies specific for histone H3, trimethyl H3K9, trimethyl H3K27, or trimethyl H4K20 with lysates prepared from HSV infected *Lmna^+/+^* and *Lmna^−/−^* MEFs at 4 hpi. The amount of ICP4 promoter sequence immunoprecipitated with each antibody was determined as described in the Materials and Methods and presented as fold enrichment relative to the cellular GAPDH gene. The data shown are the mean with error bars representing the standard error of the mean for three experiments.

To further test the hypothesis that association of heterochromatin with viral promoters in HSV-infected *Lmna^−/−^* MEFs inhibited gene expression, we conducted chromatin immunoprecipitation (ChIP) experiments. The amount of HSV DNA associated with histones was measured by ChIP using antibodies specific for histone H3 or the heterochromatin markers trimethyl H3K9, H3K27, and H4K20. The immunoprecipitated DNA was quantified by real-time PCR for the *ICP4* gene transcription start site and mouse *GAPDH* gene promoter sequences [34]. The relative amounts of viral promoters associated histones bearing the different modifications were expressed as the fraction of viral promoter sequence immunoprecipitated with the specific antibody normalized to the fraction of GAPDH DNA immunoprecipitated in the same reaction. The levels of viral DNA associated with histone H3, and thus with total chromatin were less than that for GAPDH but similar for *Lmna^+/+^* and *Lmna^−/−^* MEFs (Figure 5). In contrast, there was a 65-fold increase in trimethyl H3K9, a 6-fold increase of trimethyl H3K27 and a 23-fold increase in H4K20 associated with the ICP4 promoter sequences in *Lmna^−/−^* MEFs as compared with the *Lmna^+/+^* MEFs (Figure 5).

**Figure 6:**
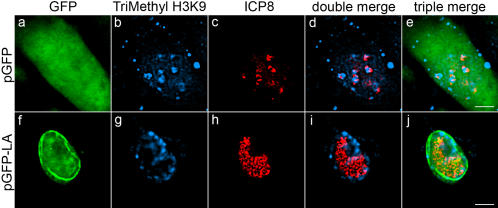
Restoration of wild-type cell phenotype by expression of lamin A in *Lmna^−/−^* MEFs. *Lmna* ^−/−^ MEFs were transfected with plasmids encoding either GFP (a–e) or GFP-lamin A (f–j). At 48 hours following transfection, cells were infected with HSV-1 at a MOI of 20 and processed for indirect immunofluorescence at 8 hpi. Antibodies for trimethyl H3K9 (b,g; blue) and ICP8 (c,h; red) were used. Double merged images of trimethyl H3K9 and ICP8 are shown in panels d and i. Triple merged images of GFP/GFP-lamin A (green), trimethyl H3K9 and ICP8 are shown in panels e and j. Scale bar = 5 µm.

### Restoration of wild-type phenotype by expression of lamin A

To confirm that association of heterochromatin with HSV DNA and replication compartments was truly the result of the lamin deficiency in *Lmna^−/−^* MEFs, we examined the distribution of heterochromatin in *Lmna^−/−^* cells transfected with plasmids encoding GFP or GFP-lamin A. In *Lmna^−/−^* cells expressing GFP, we observed co-localization of heterochromatin (HeK9Me3) with replication compartments (ICP8) (Fig. 6, panels a–e), as described above. In contrast, in *Lmna^−/−^* cells expressing GFP-lamin A, there was increased expression of ICP8, and replication compartments, as evidenced by ICP8 staining, were larger and showed an exclusion of heterochromatin (Fig. 6, panels f–j). Therefore, the observed changes in ICP8 expression, replication compartment formation, and heterochromatin distribution reverted to wild-type by expression of lamin A, arguing that the mutant cell phenotype was due to the absence of lamin A. These results in total support an important role for lamin A in reduction of heterochromatin on HSV DNA during lytic infection.

### Role of Lamin A/C in Viral Replication

**Figure 7:**
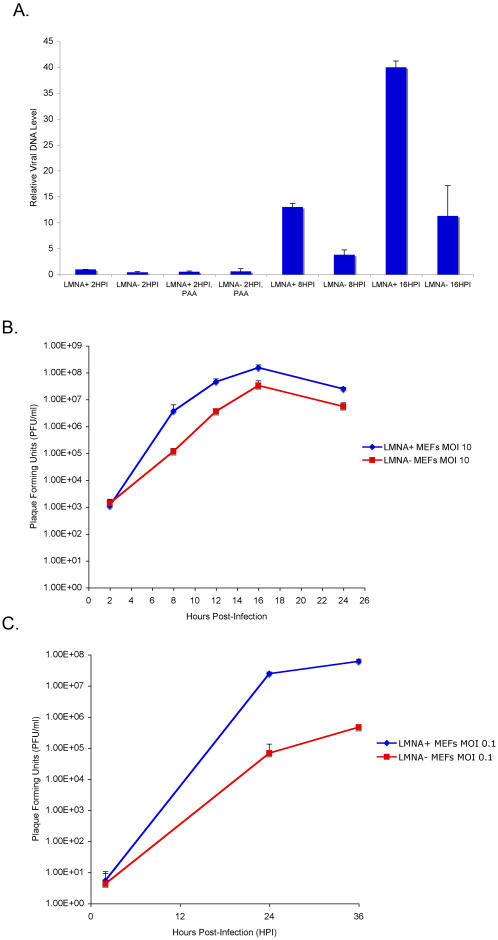
Viral DNA replication and growth defects in the absence of lamin A/C. A) Kinetics of viral DNA replication in *Lmna^+/+^* and *Lmna^−/−^* cells. DNA was extracted from whole cell lysate of mock- or HSV-infected *Lmna^+/+^* and *Lmna^−/−^* MEFs at the times indicated and quantified by real-time PCR. Relative viral DNA levels, as calculated in Materials and Methods, are shown. The error bars represent the standard deviation from three experiments. B) HSV growth curve at high MOI. *Lmna^+/+^* and *Lmna^−/−^* MEFs were infected with HSV at an MOI of 10. At the times indicated, cells and supernatant were harvested, and total infectious virus was measured by plaque assay on Vero cells. The results shown were from triplicate cultures. C) HSV growth curve at low MOI. *Lmna^+/+^* and *Lmna^−/−^* MEFs were infected with HSV at an MOI of 0.1. At the times indicated, cells and supernatant were harvested and titered on Vero cells, as described above. The results shown were from triplicate cultures.

The reduced levels of IE and E viral gene expression in the *Lmna^−/−^* MEFs predicted that the replication cycle was not being completed efficiently in these cells. Viral DNA replication was examined by real-time PCR measurement of viral DNA levels. Consistent with the reduced viral gene expression and small replication compartments, viral DNA replication was reduced by at least 3-fold in *Lmna^−/−^* MEFs compared with *Lmna^+/+^* MEFs at 8 and 16 hpi (Figure 7A). Similar viral DNA levels were seen in *Lmna^+/+^* and *Lmna^−/−^* MEFs infected in the presence of the viral DNA inhibitor phosphonacetate (PAA) for 2 hpi, arguing that the amounts of viral DNA entering the two cell types were equivalent. Viral growth was assayed by measurement of viral yields in infections of *Lmna^+/+^* and *Lmna^−/−^* MEFs at different multiplicities of infection (MOI). Viral yields were reduced by approximately 5-fold in *Lmna^−/−^* MEFs infected at an MOI of 10 (plaque-forming units per cell) as compared to *Lmna^+/+^* MEFs at 8–24 hpi (Figure 7B). Therefore, viral replication was reduced modestly in infections performed at high MOI. However, at low MOI (0.01 PFU/cell), there was an approximately 100-fold reduction in viral yield in *Lmna^−/−^* MEFs compared to *Lmna^+/+^* MEFs, arguing that the magnitude of the requirement for lamin A/C in HSV replication was multiplicity-dependent.

**Figure 8:**
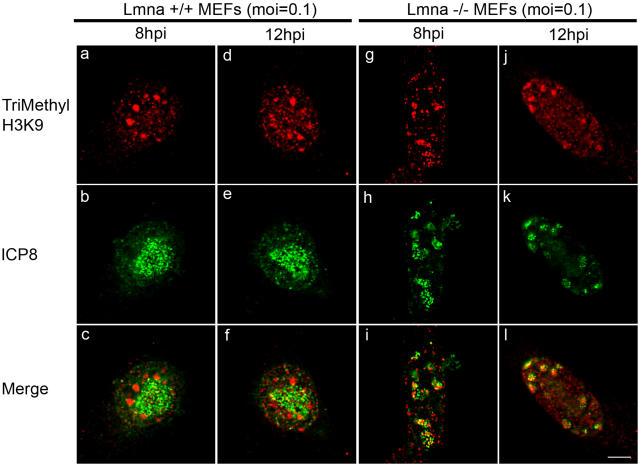
Replication compartment formation and heterochromatin distribution at low multiplicity of infection. *Lmna^+/+^* (a–f) and *Lmna^−/−^* (g–l) MEFs were infected with HSV-1 at an MOI of 0.1 and fixed at 8 hpi (a–c, g–i) or 12 hpi (d–f, j–l). Fixed cells were processed for indirect immunofluorescence using antibodies specific for ICP8 (b, e, h, k; green) and trimethyl H3K9 (a, d, g, j; red). Merged images are shown in the bottom row. Scale bar = 5 µm.

To ensure that the chromatin phenotype was also observed at low MOI, we examined replication compartment formation and heterochromatin distribution in cells infected at low MOI (0.1). At 8 and 12 hours postinfection in *Lmna^+/+^* cells, replication compartments were observed that nearly filled the infected cell nucleus and heterochromatin was marginalized along the inner nuclear membrane (Fig. 8, panels a–f). In contrast, at 8 and 12 hours postinfection in Lmna^−/−^ cells, replication compartments were small and co-localized with heterochromatin (Fig. 8, panels g–l). Therefore, lamin A/C is needed for replication compartment formation and heterochromatin exclusion at both low and high MOI's of infection but plays a more essential role at low MOI.

## Discussion

Heterochromatin is associated with the nuclear lamina, and A-type lamins have been shown to promote the maintenance of heterochromatin in mammalian cells. Thus, it is believed that the nuclear lamina is the site of heterochromatin maintenance. However, little is known about the sites or structures involved in modulation of heterochromatin. In this study we find that the A-type lamins are required for targeting of herpes simplex virus genomic complexes to the periphery of the infected cell nucleus and for preventing or reducing heterochromatin on the viral immediate-early lytic gene promoters. This raises the potential of a broader role for the nuclear lamina in the regulation of both euchromatin and heterochromatin. We propose that the nuclear lamina is a platform for the organization of chromatin remodeling and histone modification enzymes that regulate both euchromatin and heterochromatin. In HSV-infected cells, viral regulatory proteins shift the activity of these chromatin regulatory complexes to prevent assembly of or reduce heterochromatin on the viral genome so that optimal viral gene transcription can occur.

During lytic infection, only limited amounts of nucleosomes are associated with viral DNA [32],[33]. Furthermore, the histones that are associated with viral DNA bear euchromatic modifications [27],[35]. Viral gene products are believed to play a role in regulating histone association and chromatin modification on HSV DNA [1],[24]. In this study we have shown that the host nuclear lamin A/C gene products are required for histone modifications that occur on the *ICP4* gene promoter. We speculate that viral proteins, such as VP16 and ICP0, function on the nuclear lamina or in the nucleoplasmic lamin to organize enzymatic complexes that carry out euchromatic modifications of histones on the HSV genome.

### Targeting of Viral Genomes to the Nuclear Periphery

We have demonstrated that the type A lamins are required for targeting of the HSV genome to the nuclear periphery for assembly of the early replication compartments, as shown previously at early times of infection [4] and in cells along the edge of a plaque [5],[6]. Localization to the nuclear periphery is correlated with reduced levels of heterochromatin on viral IE gene promoters, arguing that viral DNA located at the nuclear periphery is protected from chromatin silencing by the host cell machinery. The HSV VP16 virion protein and the ICP0 IE protein have been shown to play roles in promoting the acetylation of histone H3 on HSV DNA. ICP0 is not required for localization of viral genomes to the nuclear periphery [6], but there is no information about VP16 as yet. Thus, the viral and cellular proteins involved in tethering HSV DNA on the nuclear periphery remain to be defined. Also, the stage in viral replication at which the HSV genome associates with the nuclear lamina or nuclear periphery is not known. Association of the viral genome with the nuclear lamina could occur at the time of IE gene transcription, E gene transcription or initiation of viral DNA replication, although our data and those of others [6] argue that this may occur at or before IE gene transcription.

Replication of the genomes of RNA viruses in the cytoplasm has also been proposed to occur on a surface but in that case on membranes (reviewed in [36]). Thus, nuclear DNA viruses may use the nuclear lamina and inner nuclear envelope as a platform for replication while cytoplasmic RNA viruses use cytoplasmic membranes as a platform for replication. It has been proposed that these surfaces provide a two-dimensional lattice or platform for assembly of replication complexes [37]. The nuclear lamina may play an additional role in providing a platform for recruitment of viral DNA as well as chromatin-modifying enzymes that keep the viral genome in an active chromatin conformation.

### Role of A-Type Lamins in HSV Replication

We found that the requirement for lamin A by HSV replication was multiplicity-dependent in that the reduction of replication in *Lmna^−/−^* cells was about 5-fold at high MOI (10 PFU/cell) while at low MOI (0.01 PFU/cell), the reduction in *Lmna^−/−^* cells was approximately 100-fold. Defects in replication compartment formation and heterochromatin association with replication compartments were observed under both conditions; thus, we believe that A-type lamins exert similar effects on viral replication at low and high MOI. At high MOI, however, the virus can circumvent the heterochromatin block. It is conceivable that at high MOI the large number of input viral genomes titrates out the finite amount of histones in the infected cell and the genomes are transcribed. Alternatively, at high MOI, the increased numbers of viral genomes eventually encounter histone-modification enzymes by less efficient means than in the assemblies located on the nuclear lamina. It is worth noting that the replication requirement for ICP0, which inhibits histone deacetylases [38], is also multiplicity-dependent [39],[40]. Therefore, the viral and cellular functions that HSV uses to prevent chromatin silencing appear to be more important at lower MOI's.

### Role of the A Type Lamins in both Heterochromatin and Euchromatin Regulation

Previous studies have largely documented a role for the A type lamins in maintenance of heterochromatin. Mutations in the human *LMNA* gene lead to premature aging and progressive loss of heterochromatin [16],[17], while immortalized mouse embryonic fibroblasts from *Lmna* ^−/−^ knockout mice exhibit alterations in nuclear envelope integrity, mislocalization of lamin-binding proteins, and reduced peripheral heterochromatin [18],[19]. In contrast, our results argue that type A lamins are necessary for preventing assembly or for removal of heterochromatin on HSV IE genes during lytic infection. Although these results may seem to be inconsistent, we propose that lamin A can serve as a platform for the organization of enzyme complexes that, under the appropriate conditions, can lead to heterochromatin or euchromatin formation on DNA sequences associated with the lamina. We further hypothesize that during HSV lytic infection viral gene products act to shift the balance towards euchromatin through the assembly of chromatin and enzyme complexes on viral lytic genes associated with the nuclear lamina that lead to euchromatic modifications of histones. In contrast, during latent infection the HSV latency-associated transcript promotes the assembly of heterochromatin on viral DNA during latent infection [34]. Thus, by regulating the type of chromatin on the viral chromosome, HSV determines whether it will undergo a productive or latent infection in different cell types. Further studies should determine the precise mechanism by which the nuclear lamins are exploited by HSV to keep its genome transcriptionally active during productive infection. These studies should provide the basis for mechanisms operative on cellular chromatin as well.

## Materials and Methods

### Cells and virus

Immortalized *Lmna* ^−/−^ murine embryonic fibroblasts (MEFs) and litter-matched *Lmna* ^+/+^ control MEFs were provided by Brian Kennedy, University of Washington [18]. Cells were grown in Dulbecco's modified Eagle medium (DMEM; Gibco) supplemented with 5% fetal bovine serum (FBS)+5% bovine calf serum (BCS), 2 mM L-glutamine, 100 U/ml penicillin and 100 µg/ml streptomycin at 37°C in 5% CO_2_. Wild type HSV-1 KOS strain virus was grown and titrated on Vero cells as described previously [8] and used for infections at the multiplicity of infection (MOI) as described. Cells were seeded one day before infection. Virus was diluted in phosphate-buffered solution (PBS) containing 0.1% glucose and applied to cells for 1 h at 37°C. The cells were washed three times for 30 seconds with an acid wash buffer (135 mM NaCl, 10 mM KCl, 40 mM citric acid buffer, pH 3), and washed with DMEM before incubation in DMEM-1% FBS medium at 37°C for the indicated periods of time. For viral growth curve experiments, HSV-1 KOS was used to infect *Lmna* ^+/+^ and *Lmna* ^−/−^ MEFs at a multiplicity of infection (MOI) of 10 or 0.01 PFU/cell. At 1 hpi, cells were washed three times for 30 seconds with acid wash buffer before incubation in DMEM plus 1% FBS for the indicated time period.

### Immunofluorescence Microscopy

*Lmna* ^+/+^ and *Lmna* ^−/−^ MEFs were seeded at 1×10^5^ cells/well on glass coverslips in 24-well plates overnight at 37°C prior to infection at the indicated MOI for immunofluorescence experiments as described previously [34]. Cells were incubated with antibodies specific for histone H1 (Upstate), histone H3K9 (Abcam), histone H4K20 (Abcam), heterochromatin protein 1α (Cell Signal Technology), HSV-1 ICP4 4040II rabbit polyclonal (Kent Wilcox), HSV-1 ICP4 mouse monoclonal (Abcam), HSV-1 ICP8 mouse monoclonal 39S [41], or HSV-1 ICP8 rabbit polyclonal 3-83 [42]. Secondary antibodies conjugated to Alexa 594, Alexa 488, and Alexa 350 dyes and prolong antifade reagent were obtained from Molecular Probes Inc. Cells were imaged on an Axioplan 2 microscope (Zeiss) with a 63× objective and Hamamatsu CCD camera (model C4742-95). Images were deconvolved using the inverse filter algorithm in the Axiovision (Rel.4.5) software.

### Transfection Method and Plasmids

Plasmid pGFP-LA, kindly provided by D.M. Gilbert, contains lamin A cDNA cloned into the pEFGP-C1 expression vector [43]. pEGFP-C2 (Clontech) described as pGFP for simplicity, was used for the expression of GFP. Three days prior to infection, *Lmna* ^−/−^ MEFs were seeded at 5×10^5^ cells per well in a 24-well plate with glass coverslips. Transfections were performed on day two, using Genejuice (Novagen) and 0.5 µg of plasmid pGFP-C2 or pGFP-LA DNA per well diluted in Optimem (Invitrogen) and 1% DMEM media without antibiotics. At day 4, or 48 hrs post-transfection, cells were infected with HSV-1 at an MOI of 20. Cells were fixed for immunostaining at 8 and 12 hpi and processed as described above.

### Hybridization Probes and Plasmids

Plasmid vectors pCI-ICP27 [44] and pBS-ICP8 (Kevin Bryant, unpublished results) were used to generate hybridization probes for the ICP27 and ICP8 mRNAs, respectively. The plasmid inserts were labeled with ^32^P dCTP (Perkin-Elmer) using Ready-To-Go DNA labeling beads (Amersham). Unincorporated nucleotides were removed from the probe using a Microspin G-50 column (GE Healthcare).

### RNA Isolation and Northern Blotting

*Lmna* ^+/+^ and *Lmna^−/−^* MEFs were either mock-infected or infected with HSV at an MOI of 20. RNA was extracted using 1mL of Trizol LS reagent (Invitrogen) per 100 mm dish. For Northern blotting, 10 µg of RNA was denatured in a solution of 50% formamide, 1.1 M formaldehyde, and 1mg/ml ethidium bromide and subjected to electrophoresis in a 10% agarose gel containing 1% formaldehyde in 1× MOPS buffer as described previously [45]. The RNA was transferred to a nitrocellulose membrane overnight in 20× SSC. The blot was incubated in QuickHyb solution (Stratagene) for 15 minutes at 68°C and then in a solution containing ^32^P labeled probes and 20 mg/ml denatured salmon sperm DNA at 68°C for 1 h. The blot was washed twice with 2× SSC-0.1% SDS for 15 minutes at 60°C and once with 0.1×SSC-0.1% SDS for 30 minutes at 60°C. The blots were exposed to a phosphorimager screen (Amersham) overnight.

### SDS-PAGE and Western Blotting

*Lmna* ^+/+^ and *Lmna^−/−^* MEFs were either mock-infected or infected with HSV-1 at an MOI of 20. At 4 and 8 hpi, cells were harvested in Laemmli sample buffer containing one protease inhibitor cocktail tablet (Roche) per 10 ml and boiled for 5 minutes. Aliquots of whole cell lysates were subjected to sodium dodecyl sulfate-polyacrylamide gel electrophoresis (SDS-PAGE), and proteins were electrically transferred to a nitrocellulose membrane. Membranes were blocked with a solution of 5% milk in PBS for an hour at room temperature and washed three times for 5 minutes in Tris-buffered saline with Tween-20 (TBST). Membranes were incubated for 2 hours at room temperature with antibodies specific for ICP0 (1∶1000), ICP4 (1∶1000), ICP8 (3-83; 1∶5000), ICP27 (1∶10,000), lamin A/C (1∶5000) or actin (1∶1000) diluted in TBST. Membranes were washed three times for 5 minutes in TBST prior to a two-hour incubation at room temperature with secondary antibodies conjugated to horseradish peroxidase diluted 1∶1000. Horseradish peroxidase signal was detected using chemiluminescence reagents (ECL; Amersham) and luminescence was detected using X-ray film (Kodak).

### Chromatin Immunoprecipitation

L*mna* ^+/+^ and L*mna* ^−/−^ MEFs were seeded at 3×10^6^ cells per 100 mm dish overnight at 37°C and were either mock infected or infected with HSV-1 at an MOI of 20. At 4 hpi cells were fixed with formaldehyde (final concentration 1% v/v) and fixation stopped with glycine (125 mM) [35]. The cells were collected by centrifugation, resuspended in lysis buffer (1% SDS; 10 mM EDTA; 50 mM Tris-HCl, pH 8.1), and incubated on ice for 10 minutes. The cell lysates were sonicated to shear DNA into lengths of ∼400 bp. The sheared chromatin was diluted 10-fold in radioimmunoprecipitation assay (RIPA) lysis buffer (0.1% SDS/1% sodium deoxycholate/150 mM NaCl/10 mM Na_2_PO_4_/2 mM EDTA/1% Nonidet P-40) with protease inhibitors. The diluted chromatin was pre-cleared with protein A agarose beads (Upstate) for 2 hours at 4°C with rotation followed by centrifugation. An aliquot (1%) of each chromatin supernatant was reserved as the input sample. The chromatin supernatant was incubated with 2.5 µg of antibody specific for histone H3 (Abcam) or 5 µg of antibodies specific for histone H3 lysine 9 trimethyl (Abcam), histone H3 lysine 27 trimethyl (Upstate), or histone H4 lysine 20 trimethyl (Abcam) at 4°C with rotation. An aliquot was incubated without antibody as a control to determine background binding. Immunocomplexes were collected by incubation with protein A agarose beads for 30 minutes at 4°C with rotation. The beads were washed three times for 5 minutes at room temperature with a low salt wash buffer (150 mM NaCl; 20 mM Tris-HCl, pH 8.1; 2 mM EDTA; 1% Triton X-100; 0.1% SDS) with protease inhibitors, followed by one wash for 5 minutes with a high salt buffer (500 mM NaCl; 20 mM Tris-HCl, pH 8.1; 2 mM EDTA; 1% Triton X-100; 0.1% SDS) with protease inhibitors. Immunocomplexes were eluted by incubation at 65°C for 30 minutes and room temperature for 15 minutes with fresh elution buffer (1%SDS; 0.1 M NaHCO_3_). Crosslinks were reversed by incubation for 4 hours at 65°C with a final concentration of 0.2M NaCl. The eluates were incubated with proteinase K, and DNA was purified by phenol: chloroform extraction, ethanol precipitation, and used as a template for real-time PCR.

### Real-Time PCR Analysis

Real-time PCR was performed by using SYBR Green and an ABI Prism 7700 sequence detection system (Applied Biosystems) as described previously [34]. PCR reactions were conducted for 40 cycles (30 s at 95°C, 60 s at 60°C) in duplicate. Mouse GAPDH Primers were previously described [34]: (GeneBank accession no. NML008084 nucleotides 781–900: 5′-CAATGT-GTCCGTCGTGGATCT-3′ and 5′-T TGAAGTCGCAGGAG-ACAACC-3′) and ICP4 gene transcript (nucleotides: 131105-131160: 5′-GCCGGGGCGCTGCTTGTTCTCC-3′ and 5′-CGTCCGCCGTCGCAGCCGTATC-3′). The amount of DNA precipitated in the ChIP assays was quantified by comparison with a standard curve, which was obtained by running a 10-fold dilution series of ICP4 or mGAPDH DNA. The amount of DNA in the no antibody control was subtracted from the amount immunoprecipitated by the appropriate antibody. The fraction of ICP4 DNA immunoprecipitated compared to the input sample was normalized to the fraction of GAPDH immunoprecipitated in the same reaction, and this value is defined as fold enrichment/GAPDH.

For quantification of viral DNA replication, cells were infected as described above and the DNA extracted using the DNeasy kit (Qiagen). Aliquots of DNA (100 ng) were used for real-time PCR and the samples run in duplicate. Viral DNA levels at each time point were quantified relative to the 2 hour postinfection sample by the ΔCt method as described [34]. To determine the relative DNA content at various times, average Ct values for the ICP4 promoter primer were subtracted by the average Ct values for GAPDH. The calibrator value (HSV sample 2-hpi) was subtracted by the GADH Ct value. To obtain the ΔΔCt value, the Ct value was subtracted by the Ct value of the input time point. ΔΔCt = (Ct_test_-Ct_reference_)−(Ct_2H ICP4_-Ct_2H GAPDH_). The fold enrichment value is 2^−ΔΔCt^.

### Accession Numbers

ICP0: NC_001806; NP_044660; (GeneID: 2703390)

ICP4: NC_001806; NP_044662; (GeneID: 2703392)

ICP8 (UL29): NC_001806; NP_044631; (GeneID: 2703458)

ICP27 (UL54): NC_001806; NP_044657; (GeneID: 2703426)

Mouse GAPDH: NC_000072; NP_032110 (GeneID: 14433)

Human Lamin A/C: NC_000001; NP_733821; (GeneID: 4000)

Mouse Lamin A/C: NC_000069; NP_001002011; (GeneID: 16905)
